# The Attitude of the Public

**Published:** 1921-08-13

**Authors:** 


### THE PROBLEM OF THE INSANE.

### I.—The Attitude of the Public.

There are certain questions which seem to have attached to them a strong emotional complement; that is, they can never be discussed without much personal bias, and the language used is frequently acrimonious. The theory of Relativity or the practical bearings of the fifth proposition of Euclid leave most of us calm and cold; but let us discuss theology, or tariffs, or Ireland—we are moved to the depths of even our autonomic systems! Often enough it is a matter of knowing the facts or of not knowing them. What we do not know we make up for by a discharge of emotional energy. Our acquisition of knowledge is generally so slipshod that we get into the habit of learning a few facts—or accepting them as facts on the authority of a more or less competent authority—and padding out our mental content by means of inference and analogy. It is too much trouble to study diligently; and, even though we speak of our time as one of undue specialisation, the fact remains that most of us have a mere smattering of knowledge of a variety of subjects.

Nowhere is this more clearly to be seen than in regard to psychology, normal and morbid, and to all that thereto pertains. Consider the past hundred and thirty years in relation to such matters. What would the average person say of that period? Would he know even the name of Gall, whose researches into brain structure and function were so thorough? The only association would be that of phrenology, the subsidiary and theoretical part of Gall's contribution to knowledge. But that is more spectacular, and therefore has the greater appeal. So it goes on: the men of solid achievement are comparatively unknown. Fortunately, their work has steadily been incorporated into the fabric of our social system, and has not, therefore, been lost. It is the popular view that most abuses have been eliminated because of the clamour of the Press or of the writers of fiction. Sometimes this may have been so; and in any case one would not wish to disparage their good intentions. On the other hand, it is well to remember that they have not invariably stood for progress, and that reforms have been carried out in spite of their reactionary views. How much help did Pinel, Tuke, Gardiner Hill, and Conolly receive from those sources? Yet these were the men who initiated the reforms in the treatment of the insane. Often enough the reward for having made such efforts to bring about an advance is the criticism: "But why have you not gone further?" And as it has been in the past so is it now. For every article or book dealing with the advance that has been made in the care and treatment of the insane there are, at a low estimate, ten which lay stress upon the "scandals" and "abuses." Even the word of a lunatic is taken as sufficient evidence to bolster up charges of ill-treatment and neglect, though the person who accepts such testimony as true would not be altogether pleased were he himself found guilty by such a process. Yet this kind of thing happens time and again because, through indolence or prejudice, no attempt is made to ascertain the facts. Nor can we leave out of account the emotional complex already mentioned, which renders people nurtured on *Hard Cash* and its congeners prone to accept all too readily anything which tends to support their preconceived ideas.

It would be idle to assert that, in regard to lunacy and to asylums, everything is for the best. All those who have the interests of patients and their proper housing and treatment at heart will realise that much remains to be done. Nor do they deprecate unduly the criticisms of the candid friend. There has recently been a vast deal of excitement about the scandalous conditions prevailing in the county asylums in this country, and it is a fair inference that the private asylums are included in the ban. A certain Dr. Lomax has written a volume in which he details his experiences in two of the county asylums,* and, to judge from the comments in the Press, his experiences were decidedly unpleasant. So lurid have the headlines been that those who are practically acquainted with the facts probably came to the conclusion that here was another of those bits of special pleading which are so partisan as to be worthless. To judge Dr. Lomax by these comments—even by the extracts given—is to do him a disservice. Taken by and large, his book is a temperate statement of things as he found them during his limited experience—he worked for two years in one asylum and two months in another. They seem to have been—accepting his statements, and there appears to be no reason to impugn them—not too well managed. But to infer from what he saw that all other asylums may be judged by those in which he worked is neither logical nor fair. It is to be noted also that his experience was gained during the war, when the conditions in asylums were generally far less satisfactory than during normal times. It is fair to Dr. Lomax to state that he makes some allowance for that time of stress; nevertheless, his indictment is a serious one, and merits consideration. Doubtless it will receive attention in the proper quarters; for, in spite of his allegations of official inertia, there is not only the desire but the will to bring about further improvements in the asylum world. In any case, it is interesting to have the opinion of a medical man who has less of the ignorance and the prejudice which usually characterise those who assail the administration of asylums in *this* country.

It is quite the usual practice when criticising

* *The Experiences of an Asylum Doctor: with Suggestions for Asylum and Lunacy Law Reform.* By Montagu Lomax, M.R.C.S. (London: Geo. Allen & Unwin, Ltd. 12s. 6d. net.)

our treatment of the insane to point out how much better they do these things in other countries. It is an easy method of attack, because it is not possible for the ordinary person to see for themselves the system adopted elsewhere. Not that they would take the trouble so to instruct themselves if they had the chance—and this is said quite advisedly, and because of the attitude of the average person in regard to visiting asylums. For, while they are quite prepared to join in the clamour and the criticism, they would "rather die than live among these people." Their prejudice is to some extent justifiable. Whenever a person commits some particularly abominable act, or his conduct in other ways renders him a pest and a nuisance to his neighbours, it is habitual to remark that he must be insane, and more often than not it is a true saying. Thereupon he is sent to an asylum, and rightly so. Not even the sentimentalist would wish him to remain outside—unless he lived far enough away. Now, it may be that the patient is only exhibiting symptoms of a transient mental disorder. He may be in the preliminary stage of an attack of acute mania, and the more acute and obvious symptoms will supervene, regardless of what we may do. It is a great pity from the point of view of enlightening the ignorance of people who—to use an expressive colloquialism—talk through their hat that they cannot be made to undertake the care of one or two patients suffering in this way. Fortunately—for the patients—no such thing is possible. The tender mercies of the wicked—doctors, nurses, and attendants—are less cruel than the irritated philanthropists would be!

Those who work in asylums are well aware of the disastrous results brought about by the frequently well-meant, but more often misguided, efforts of the amateur alienist. Many patients are brought to the asylum in an exhausted or starved condition, covered with bruises, filthy, and verminous. It is not only the laity who are to blame for this. Not infrequently various new methods of mental healing have been tried—while the patient has become steadily worse. At times one feels apt to despair at the public attitude towards asylums as exemplified by this putting off the day which seems to many of them so evil. Even those who know what asylum life really is begin to think that they would be better employed in any other job rather than continue in one which is so often held up to obloquy. Nothing has been so lamentable in the storm raised by Dr. Lomax's book than the avidity with which the Press has seized upon anything that is sensational in it, while leaving out any reference to other matters with which he deals, but which have not the same likelihood of tickling the ears of the groundlings.

*(To be continued.)*

